# Orexin receptor antagonists in the treatment of insomnia associated with psychiatric disorders: a systematic review

**DOI:** 10.1038/s41398-024-03087-4

**Published:** 2024-09-14

**Authors:** Taro Kishi, Michinori Koebis, Michiko Sugawara, Yuka Kawatsu, Takehiro Taninaga, Nakao Iwata

**Affiliations:** 1 Department of Psychiatry, Fujita Health University School of Medicine, Toyoake, Aichi Japan; 2 Medical Headquarters, Eisai Co, Ltd, Tokyo, Japan

**Keywords:** Bipolar disorder, Depression

## Abstract

Insomnia is highly comorbid in patients with psychiatric disorders, including depression, bipolar disorder, and substance use disorders, and should be treated as an independent condition. Dual orexin receptor antagonists (DORAs) have been investigated as a treatment for chronic insomnia. The objective of this systematic review was to examine evidence for two DORAs, lemborexant and suvorexant, as treatments for insomnia comorbid with a psychiatric disorder. We searched PubMed, Cochrane, and Embase from their inception until January and April 2023, and included studies examining suvorexant and lemborexant for treating insomnia comorbid with psychiatric disorders. We also manually searched clinical trial registries (https://clinicaltrials.gov and https://www.umin.ac.jp/ctr). Randomized clinical trials and observational/cohort studies were included. We identified 18 studies from PubMed, Cochrane, and Embase and three studies from clinicaltrials.gov and UMIN. Of the 21 reports, four were completed/terminated randomized clinical trials, eight were ongoing clinical trials, and nine were observational studies. We identified evidence for switching from benzodiazepine receptor agonists to a DORA, or using a DORA as add-on therapy and, therefore, discuss this topic as well. Two studies examined switching to or adding on a DORA in patients being treated with a benzodiazepine receptor agonist. DORAs may be as effective and safe for treating psychiatric comorbid insomnia (for most psychiatric conditions) as they are for treating primary insomnia. However, the evidence is limited to a few small studies. Further investigation of DORAs for the treatment of comorbid insomnia in those with coexisting psychiatric conditions is warranted.

## Introduction

Insomnia has a high comorbidity rate in psychiatric disorders, including depression [1], bipolar disorder [2, 3], schizophrenia [4–8], generalized anxiety disorder [9, 10], and substance use disorders [11]. The comorbidity of insomnia is related to the severity of psychiatric symptoms [12–15]. For instance, patients with depressive symptoms and sleep disturbances may experience more intense and frequent anxiety symptoms, as well as worse cognitive and physical functioning compared with depressive patients without sleep disturbances [12].

Insomnia symptoms are also related to the prognosis and risk of recurrence of comorbid psychiatric disorders [16–20]. Patients with depression comorbid with residual sleep disorders have been reported to have a 4.8-fold risk of depression recurrence compared with those without sleep disorders [16]. Insomnia has also been observed as a prodromal symptom prior to a mood episode recurrence in patients with bipolar disorder [17], and patients with schizophrenia comorbid with sleep disorders may be at a higher risk of worsening symptoms in response to antipsychotic withdrawal [18].

Some psychotropic drugs have sedative effects, which may help alleviate insomnia symptoms [21–24]. Nonetheless, the fact that many patients with comorbid insomnia experience residual insomnia even after remission of the psychiatric disorder [24] leads to the idea that insomnia is an independent disorder from the comorbid psychiatric disorder. Indeed, the distinction between primary and secondary insomnia has been effaced in the Diagnostic and Statistical Manual of Mental Disorders, Fifth Edition (DSM-5), and the International Classification of Sleep Disorders, Third Edition. As such, psychiatric symptoms and insomnia should be treated independently in cases where they appear concomitantly [25, 26].

Current guidelines recommend the use of cognitive behavioral therapy for insomnia (CBT-I) as standard therapy in the treatment of chronic insomnia [27–29]. This is supported by a recent systematic review and meta-analysis, the authors of which also highlighted the importance of the specific treatment of insomnia in patients with a comorbid mental disorder [30]. However, despite the favorable benefit–risk balance of CBT-I, not all patients with insomnia will derive benefit from this approach, with reports that responses to CBT-I may be attenuated in insomnia patients with objective short sleep duration compared with those with normal sleep duration [31, 32]; furthermore, access to, or reimbursement for, CBT-I may be limited in some countries. Thus, pharmacotherapy also represents a major component of treatment for insomnia [33] and insomnia complicated by psychiatric disorders.

Benzodiazepine receptor agonists (BZRAs) including benzodiazepines and Z-drugs (i.e., zaleplon, zolpidem, zopiclone, and eszopiclone) [34] have, to date, comprised the primary pharmaceutical treatment approach for insomnia comorbid with a psychiatric disorder. Several randomized clinical trials (RCTs) investigating Z-drugs for depression with comorbid insomnia have been reported [35–37]. However, long-term use of BZRAs is problematic for various reasons, including dependence and withdrawal symptoms [38]. Recently, dual orexin receptor antagonists (DORAs), which have a different mechanism of action from BZRAs, were developed and launched as a treatment for insomnia. Suvorexant and lemborexant have been approved in several countries. Orexin is a neuropeptide produced by a limited number of neurons in the lateral hypothalamus that project to a wide range of brain regions, including monoaminergic and cholinergic neurons. Orexin binds to orexin receptor OX1R and OX2R on these neurons to maintain alertness. DORAs inhibit the effect of orexin by binding both types of receptors, thus reducing wakefulness. Monoaminergic and cholinergic systems are deeply implicated in the pathogenesis of psychiatric disorders. Nonclinical studies have reported that antagonism of orexin signaling reduces anxiety-like and substance dependence-like behaviors in rodents [39]. The orexin receptor antagonist seltorexant is a selective antagonist of OX2R being studied in a phase 3 trial in patients with major depressive disorder with insomnia (NCT04533529) [40].

Although DORAs are effective in insomnia disorder [41, 42], there is limited evidence for their efficacy in insomnia as a psychiatric comorbidity [43]. In 2020, we published a network meta-analysis comparing the DORAs suvorexant and lemborexant in insomnia [44]. However, the clinical studies assessed in that analysis were limited regarding how many patients with an active psychiatric condition were included. Thus, in the present study, we sought to review available clinical evidence for DORAs in the context of insomnia as a comorbid condition with a psychiatric disorder. As such, the objective of this review was to evaluate the evidence on suvorexant and lemborexant as a treatment for insomnia comorbid with a psychiatric disorder, based on RCTs and observational studies. We discuss the usefulness of DORAs for insomnia comorbid with psychiatric disorders. We also discuss the incidental evidence for switching from a BZRA to a DORA or using a DORA as an add-on to BZRA treatment, as BZRAs have been widely used for insomnia comorbid with psychiatric disorders.

## Methods

### Search strategy

A literature search for this systematic literature review was conducted to identify research studies, including completed and ongoing RCTs and observational studies, of suvorexant and lemborexant in the treatment of insomnia comorbid with psychiatric disorders. This review was registered and a review protocol is available at OSF Registries (10.17605/OSF.IO/SFNUR). The primary search was carried out in January 2023 using the PubMed, Embase, and Cochrane databases. Search strings followed the following structure: (lemborexant OR suvorexant) AND (various keywords for different mental disorders combined with ‘OR’, based on DSM-5 [25] criteria and code; see Table S1). Delirium was not included in the scope of psychiatric disorders in this study because the primary objective of many studies in patients with delirium was to investigate the effect on prevention or treatment of delirium itself, which made it difficult to evaluate the effect of the treatment on insomnia in this population [45]. PubMed and Cochrane were searched on January 19, 2023, and Embase was searched on January 24, 2023 (Fig S1). To update the search results, we conducted a second search for studies published in these databases between the date of the first systematic search and April 30, 2023 (Fig. S2).

After removing duplicates, “Chapter,” “Conference Abstract,” “Conference Paper,” “Conference Review,” “Editorial,” “Note,” and “Review” were automatically excluded based on the publication type annotation of the record (Figs. S1 and S2). In addition, trial registries, specifically clinicaltrials.gov (https://clinicaltrials.gov) and the University Hospital Medical Information Network (UMIN; https://www.umin.ac.jp/ctr), were manually searched for “suvorexant” or “lemborexant”.

### Study selection

For the study selection for analysis in this systematic review, the following inclusion criteria were applied: studies using suvorexant and lemborexant for the treatment of insomnia comorbid with psychiatric disorders; all or a majority of patients in the study had comorbid psychiatric disorders; and post hoc analyses of insomnia clinical trials with stratified analysis of patients with comorbid psychiatric disorders. Case series, reviews, meta-analyses, editorials, notes, chapters, and conference presentations were excluded.

Primary screening was conducted based on title and abstract, followed by full-text screening, by at least two independent authors (MK, YK, and TT), and any discrepancies were discussed by the two authors until an agreement on inclusion/exclusion was reached. We then manually searched and screened results from clinicaltrials.gov and UMIN, in addition to the articles identified from PubMed, Cochrane, and Embase.

## Results

Based on the first systematic search conducted on the PubMed, Embase, and Cochrane databases, 412 records were obtained after removing duplicates (Fig. S1). Following exclusion based on title and abstract, 60 full-text articles were assessed for eligibility, and 17 records were finally included. We also included three additional reports from clinicaltrials.gov and UMIN. After removing duplicates, the second systematic search yielded 41 records that were newly published; one of these records was eligible for inclusion (Fig. S2). In total, 21 reports were evaluated, including 4 completed/terminated RCTs (Table 1), 9 observational/cohort studies (Table 2), and 8 ongoing clinical trials (Table 3; 1 trial was terminated after the systematic search).

**Table 1:** Randomized clinical trials.

Drug	Study design	n	Comorbid psychiatric disorders	Treatment	Sleep measurement	Sleep-related outcomes	Safety outcomes	Reference
SUV	Double-blind RCT	37: SUV 18 Placebo 19	Trauma	SUV vs PBO, 2 weeks	ISI, PSG	ISI: significantly decreased (*p* < 0.001; no significant difference between groups) PSG: TST and REM sleep% significantly increased (*p* < 0.05, *p* < 0.01; no significant difference between groups)	Discontinued: SUV, 1 (moderate sedation, lack of efficacy)	Mellman et al. [46]
SUV	Double-blind RCT	48: SUV 23 Placebo 25	Bipolar disorder	SUV vs PBO, 1 week → SUV (all patients), 3 months	sTST, TST	sTST (1 week): no significant change from baseline in SUV group, no significant difference between groups TST (1 week): significant increase in SUV, (*p* < 0.035; no significant difference between groups) sTST, TST (3 months): no significant change from week 1	AEs: Hypersomnolence: SUV, 1 (1 week) 3 (1 week to 3 months), PBO, 1 (1 week)	NCT02527564
SUV	Open-label RCT	18: SUV 9 ESZ 9	MDD	Switch from BZs to SUV or ESZ, 4 weeks (tapering BZs in 2 weeks)	ISI, PSQI	Outcome: no significant difference between groups ISI: improved in both groups (significant improvement in ESZ: *p* = 0.04 for baseline vs both weeks 2 and 4; no significant change in SUV) PSQI: improved in ESZ (no significant change)	Discontinued due to AE, ESZ, 2	Shigetsura et al. [48]

*AE* adverse event, *BZ* benzodiazepine, *ESZ*, eszopiclone, *ISI* Insomnia Severity Index, *MDD* major depressive disorder, *PBO* placebo, *PSG* polysomnography, *REM* rapid eye movement, *RCT* randomized clinical trial, *sTST* subjective total sleep time, *SUV* suvorexant, *TST* total sleep time, *PSQI* Pittsburgh Sleep Quality Index.

**Table 2:** Observational and cohort studies.

Drug	Study design	n (N)^a^	Comorbid psychiatric disorders	Treatment	Sleep measurement	Sleep-related outcomes	Safety outcomes	Reference
SUV	Open-label prospective trial	57	Psychiatric disorders: MDD, 49.1% Bipolar disorder, 17.5% Schizophrenia, 14.0% Eating disorder, 5.3% Adjustment disorder 3.5%	SUV 7 days	sTST, sTSO, sWASO, sleep satisfaction	sTST, sTSO, sWASO, sleep satisfaction: significantly improved from baseline (*p* < 0.0001, *p* = 0.0003, *p* < 0.0001, *p* = 0.0349, respectively), severity of mental illness: not significant	Discontinued: AE, 7 Inefficacy, 2	Kishi et al. [50]
LEM	Open-label prospective trial	56	Psychiatric disorders: Schizophrenia, 16.1% MDD, 21.4% Bipolar disorder, 32.1% Anxiety disorder, 3.6% Adjustment disorder, 14.3% Eating disorder, 8.9%	LEM 7 days	sTST, sTSO, sWASO, sleep satisfaction	sTST, sTSO, sWASO, sleep satisfaction, severity of mental illness: significantly improved from baseline (*p* < 0.0001, *p* < 0.0001, *p* < 0.0001, *p* < 0.0001, *p* = 0.0006, respectively)	Discontinued: AE, 5 Inefficiency, 1 Improved, 1	Kishi et al. [52]
SUV	Open-label prospective trial	38 (41)	Psychiatric disorder, 38: Schizophrenia, 11 MDD, 5 Bipolar disorder, 3 Anxiety disorder, 2	SUV 4 weeks	PSQI	Responder (PSQI ≥ 3 point improvement): *n* = 19/38	Discontinued: Somnolence, 1 Liver failure, 1	Izuhara et al. [49]
SUV	Open-label prospective trial	40	Schizophrenia, 12 Mood disorder, 16 Neurotic and stress-related disorder, 3	SUV 8 weeks	PSQI	Sleep quality scores: Significantly decreased (Week 2, *p* < 0.01; Week 4, *p* < 0.01) Sleep duration, habitual sleep efficacy, global scores: Significantly decreased (Week 4, *p* < 0.05)	Discontinued: Somnolence, 1 Fatigue, 1	Nakamura et al. [51]
SUV	Prospective cross-sectional study (post-marketing surveillance)	1007 (3248 in SAS)	Psychiatric disorders, 1007: Depression, 508 Anxiety disorder, 211 Schizophrenia, 92 Manic depression, 84	SUV < 6 months	Comprehensive assessment of sleep effect by physician	Improved patient rate: With psychiatric comorbidity, 70.6% (534/756) Without psychiatric comorbidity, 75.9% (1246/1642)	ADRs: With psychiatric comorbidity, 14.3% (144/1007) Without psychiatric comorbidity, 7.6% (166/2182)	Asai et al. [57]
LEM	Retrospective cohort study	649	Psychiatric disorders, 90.9% Schizophrenia, 8.3% Depressive disorder, 24.8% Anxiety disorder, 14.8% Trauma/stressor-related disorder, 9.9% Bipolar disorder, 8.8%	LEM 1–8 weeks	CGI-I	Responder rate (CGI-I score, 1–3): 64.5%	AEs: Somnolence, 5.1% Headache, 2.6% Nightmare, 4.9% Tiredness or malaise, 2.6%	Katsuta et al. [56]
SUV	Retrospective cohort study	91	Schizophrenia, 35% Mood disorder, 32% Dementia, 13%	―	―	Continued SUV (3 years), 29 (32%)	Discontinued: Inefficacy, 31 Somnolence, 6 Headache, 1 Improvement, 6 Other, 2	Murata [54]
SUV	Retrospective cohort study	228	Psychiatric illness, 167: Schizophrenia, 26 Bipolar disorder, 45 MDD, 59 Others, 37	Prescribe SUV for patient receiving BZs Switch group: decrease dose or discontinuation of BZs Add-on group: add SUV with BZs	―	BZ dose (diazepam equivalence): Switch group, 8.5 ± 5.8 → 3.9 ± 4.9 (*p* < 0.001) BZ discontinuation: Switch group, 46.2%	SUV discontinued: Add-on group, 49 (45.0%) Inefficacy, 18 Intolerability, 24 Somnolence, 12 Switch group, 29 (24.4%) Inefficacy, 18 Intolerability, 9 Somnolence, 5	Hatano et al. [58]
LEM	Retrospective cohort study	150	Schizophrenia, 2 Depression, 59 Bipolar disorder, 24 Anxiety disorder, 53	LEM 6 months	AIS CGI-I	AIS: 6.6 ± 3.7 → 3.9 ± 3.3 significantly improved (*p* < 0.01) CGI-I: 3.2 ± 0.8 (6 months)	Discontinued: Improved, 21 Patient’s decision, 2 Lack of efficacy, 4 Sleepiness, 5 Fatigue, 1 Nightmares, 1	Suzuki et al. [55]

*ADR* adverse drug reaction, *A*E adverse event, *AIS* Athens Insomnia Scale, *BZ* benzodiazepine, *CGI-I* Clinical Global Impression of Improvement, *LEM* lemborexant, *MDD* major depression disorder, *PSQI* Pittsburgh Sleep Quality Index, *sTSO* subjective time to sleep onset, *sTST* subjective total sleep time, *SAS* safety analysis set, *SUV* suvorexant, *sWASO* subjective wake after sleep onset.

^a^Number of patients: with comorbid psychiatric disorder (total patients).

**Table 3:** Ongoing clinical trials.

Drug	Study design	Comorbid psychiatric disorders	Intervention	ID (Study end)
SUV	Open-label, prospective cohort study	Substance use disorders	SUV is administered for 7 days. Primary assessment is change in TST measured by actigraph. Secondary assessments include craving for dependent substances, cortisol, and dependence on SUV.	NCT03412591 (Nov. 2023)
SUV	RCT vs placebo	Alcohol dependence	Patients with insomnia with alcohol dependence will be treated with SUV or placebo for 6 months and their sleep status, ISI, PSQI assessed by PSG, craving for alcohol.	NCT03897062 (Jul. 2022)
SUV	RCT vs placebo	PTSD	SUV is administered in veterans with PTSD as a flexible dose design (2-week titration followed by a 10-week steady-dose phase) to test SUV’s efficacy and safety in trauma-related sleep disturbance.	NCT03642028 (Dec. 2024)
SUV	RCT vs placebo	Opioid dependence	Patients with insomnia with opioid dependence will be treated with SUV or placebo for 8 weeks and their sleep status (TST, total WASO) assessed by actigraph; stress scores will be evaluated.	NCT04287062 (Nov. 2024)
LEM	RCT vs placebo	Opioid use disorder	In people with opioid use disorder, LEM will be tested when used in combination with opioids to assess effects on sleep problems.	NCT04818086 (Apr. 2023)
SUV	RCT vs placebo	MDD	SUVs or placebo in combination with antidepressant treatment for 6 weeks in patients with depression with insomnia. Primary assessment is TST; secondary assessments include ISI and WASO.	NCT02669030 (Dec. 2019)
LEM	RCT vs placebo	Schizophrenia	In patients with schizophrenia with insomnia treated with LEM 5 mg or placebo for 14 days, sleep variables assessed by actigraph are compared.	UMIN000049310 (Terminated)
LEM	RCT vs placebo	Alcohol dependence	Patients with insomnia with alcohol dependence will be randomly allocated to naltrexone plus LEM for 4 weeks or naltrexone plus placebo. The primary assessment will be craving for alcohol and secondary assessments will be sleep quality, insomnia severity, and sleep status as measured by actigraph.	NCT05458609 (Aug. 2025)

*ISI* Insomnia Severity Index, *LEM* lemborexant, *MDD* major depression disorder, *PSG* polysomnography, *PSQI* Pittsburgh Sleep Quality Index, *PTSD* post-traumatic stress disorder, *RCT* randomized clinical trial, SUV suvorexant, *TST* total sleep time, *WASO* wake after sleep onset.

### Randomized clinical trials

Among the four RCTs identified, there were three placebo-controlled trials with suvorexant, and one open-label trial in which a benzodiazepine-based sleeping medication was switched to suvorexant or eszopiclone (Table 1). In the placebo-controlled trial of patients with post-traumatic stress disorder (PTSD)-related insomnia, participants were enrolled if they had insomnia that began or was significantly exacerbated following a DSM-5 Criterion A trauma within 10 years of enrollment (*n* = 37, suvorexant *n* = 18, placebo *n* = 19) [46]. Of the enrolled patients, 11.1% in the suvorexant group and 21.1% in the placebo group had maintained the PTSD diagnostic criteria at the time of enrollment [46]. The Insomnia Severity Index score significantly decreased regardless of treatment group, and there were no differences between treatment groups. Additionally, polysomnography showed significant increases in total sleep time (TST) and the percentage of rapid eye movement sleep across all subjects, albeit no significant differences were detected between groups. There was no significant improvement in the Nightmare Severity Scale, which may be partly attributable to low baseline values. Regarding safety, of the 41 patients in the safety analysis in the study of patients with PTSD-related insomnia [46], one in the suvorexant group discontinued due to moderate somnolence, and another in the suvorexant group developed mild somnolence, which improved without discontinuation. Neither nightmares nor rapid eye movement-related parasomnias were observed.

In a study of patients with insomnia associated with bipolar disorder who were on treatment (*n* = 48; *n* = 23 in the suvorexant group and *n* = 25 in the placebo group), there were no differences between groups in either subjective TST (sTST) or actigraphy-measured TST at 1 week [47]. Regarding safety, somnolence was observed as an adverse event (AE) in one patient in the suvorexant group and in one patient in the placebo group by the first week of treatment; somnolence was also observed in three of the 46 patients in the extended period of suvorexant treatment up to 3 months [47]. There was another study of suvorexant in patients with insomnia and bipolar disorder, but it was terminated due to COVID-19 (NCT03764683, not shown in Table 1).

The fourth, open-label trial with results available was carried out in patients with major depressive disorder with comorbid insomnia and is discussed below [48].

### Prospective and retrospective observational and cohort studies

No prospective cohort studies using DORAs have been reported in patients with comorbid insomnia with any specific psychiatric disorder, but there have been studies reported in patients with comorbid insomnia with psychiatric disorders more generally (Table 2), including three with suvorexant [49–51], and one with lemborexant [52]. In addition, four observational retrospective cohort studies have been conducted, two with suvorexant [53, 54] and two with lemborexant [55, 56]. One post-marketing study was also reported with suvorexant [57].

Kishi et al. conducted two open-label, prospective interventional studies with similar protocols, one with suvorexant and one with lemborexant [50, 52]. The 2019 study with suvorexant examined 57 psychiatric patients with insomnia symptoms and found significant improvements in sTST, subjective time to sleep onset, subjective wake time after falling asleep, and patient sleep satisfaction during the first week of suvorexant treatment [50]. Similarly, the 2022 study with lemborexant examined 56 psychiatric patients with insomnia symptoms and also found significant improvements in sTST, subjective time to sleep onset, subjective wake time after falling asleep, and patient sleep satisfaction, with consistent effects regardless of whether the patient had received a prior baseline dose of sleep medication [50, 52]. Furthermore, subjective visual analog scale scores for severity of mental illness (0 = extremely ill, 10 = no symptoms) improved by 0.53 ± 2.07 and 0.93 ± 2.15 points after 7 days of suvorexant or lemborexant treatment, respectively, with the latter showing a significant improvement compared with baseline [50, 52]. AEs in the suvorexant study with 57 patients included somnolence (28.8%), fatigue (11.5%), and nightmares (5.8%), and seven discontinuations were caused by AEs (somnolence [*n* = 3], somnolence and fatigue [*n* = 1], fatigue [*n* = 1], nightmares [*n* = 1], and vomiting [*n* = 1]) and inefficacy (*n* = 2) [50]. In the lemborexant study with 56 patients, fatigue (8.9%) and somnolence (7.1%) were observed, and discontinuation was caused by an AE in five cases (dizziness [*n* = 2], agitation [*n* = 1], nightmares [*n* = 1], and sleep paralysis [*n* = 1]) and inefficacy in one case [52].

Katsuta et al. conducted a retrospective observational study to examine the real-world safety and effectiveness of lemborexant in insomnia comorbid with psychiatric disorders [56]. In total, 649 patients with different psychiatric disorders were treated with lemborexant for 1 to 8 weeks. Lemborexant response was assessed by the Clinical Global Impression of Improvement scale, and 64.5% of patients were considered responders based on improvements in insomnia symptoms. The proportion of responders was ≥60% for most types of psychiatric disorders, including neurodevelopmental disorders (67.7%), bipolar disorders (71.9%), depressive disorders (67.1%), anxiety disorders (64.6%), trauma- and stressor-related disorders (62.5%), and neurocognitive disorders (62.9%). The safety profile of lemborexant was favorable, with the majority of responders (94.7%) reporting no AEs. The most frequent AEs in the total population included somnolence (5.1%), headache (2.6%), nightmare (4.9%), and tiredness or malaise (2.6%).

Izuhara et al. examined response to suvorexant in patients with insomnia with a comorbid psychiatric disease, and 20 of 41 patients (49%) in the study population responded to suvorexant with a Pittsburgh Sleep Quality Index (PSQI) improvement of ≥3 points at 4 weeks; of the 38 patients with a psychiatric comorbidity, 19 (50%) responded to suvorexant [49]. In addition, all five patients with depression and 7 of the 13 patients (54%) with schizophrenia and schizoaffective disorder responded to suvorexant [49]. In another study, Nakamura et al. reported that the global PSQI score significantly decreased from baseline at Week 4 (12.21 ± 0.85 to 9.14 ± 0.79, *p* < 0.05) [51] as a result of suvorexant treatment in patients with psychosis who also had sleep disturbances (*n* = 40). The change in comorbid psychiatric symptoms also improved: anxiety symptoms (Generalized Anxiety Disorder-7 scale) improved from 10.52 ± 1.19 to 5.72 ± 1.10 and depressive symptoms (Patient Health Questionnaire-9) significantly improved from 13.97 ± 1.44 to 8.79 ± 1.25 (*p* < 0.01) at week 4.

A prospective registry study (post-marketing surveillance) evaluated the efficacy of suvorexant in 2439 patients with insomnia, of whom 756 had a comorbid psychiatric disorder [57]. The rate of improvement in insomnia symptoms based on physician judgment was 70.6% (534/756) for patients with comorbid psychiatric disorders and 75.9% (1246/1642) for patients without psychiatric disorders. By type of mental illness, the respective improvement rates were 68.4% and 74.5% for patients with and without schizophrenia, 72.5% and 74.6% for patients with and without depression, 59.4% and 74.7% for patients with and without manic-depressive illness, and 72.1% and 74.4% for patients with and without anxiety disorders, with no specific disorder significantly associated based on a multiple regression analysis. In addition, the incidence of adverse drug reactions in patients with psychiatric disorders was 14.3% (144/1007), which was higher than the incidence of 7.6% (166/2182) in patients without psychiatric disorders. The respective incidences of adverse drug reactions by psychiatric disorder were 14.1% and 9.6% for patients with or without schizophrenia, 13.8% and 8.9% for patients with or without depression, and 12.8% and 9.5% for patients with or without anxiety disorders. The major adverse drug reactions in patients with comorbid psychiatric disorders included somnolence, insomnia, dizziness, malaise, and nightmare, which were also common in patients without psychiatric disorders. It was not mentioned if there were adverse drug reactions specific to patients with psychiatric disorders. Approximately half (1671/3248; 52.4%) of the patients discontinued treatment for reasons including improvement (17.1%), ineffectiveness (10.9%), lack of effect (4.9%), and AEs (8.0%).

A retrospective cohort study of 150 patients with sleep disorders and comorbid psychiatric disorders presenting to a psychiatric clinic who were treated with lemborexant for 6 months showed significant improvements in total Athens Insomnia Scale score (*p* < 0.01), with 21 patients achieving remission and discontinuing the treatment [55]. Other reasons for discontinuation included lack of effect (*n* = 4), somnolence (*n* = 5), fatigue (*n* = 1), and nightmares (*n* = 1).

### Switching from benzodiazepines to DORA

We found a few studies that investigated outcomes for patients switching from benzodiazepines to a DORA.

In an open-label RCT of patients with insomnia with comorbid major depressive disorder (*n* = 18) who did not respond adequately to benzodiazepines and then switched to either suvorexant (*n* = 9) or eszopiclone (*n* = 9), the Insomnia Severity Index score declined after switching similarly in both groups, and there were no significant differences between the groups (Table 1) [48].

In a retrospective cohort study of patients with insomnia (*n* = 228) taking benzodiazepines, the retention rates of suvorexant treatment were confirmed for both a “Switch” group who substituted all or a part of benzodiazepines with suvorexant and an “Add-on” group who did not reduce the benzodiazepine dose at suvorexant initiation (Table 2) [58]. Among all study participants, 73.2% had comorbid psychiatric disorders (depression 25.9%, bipolar disorder 19.7%, schizophrenia 11.4%). The discontinuation rate was 24.4% in the Switch group and 45.0% in the Add-on group. The main reasons for discontinuation in the Switch group were inadequate efficacy (62.1%), intolerance (31.0%), and somnolence (17.2%), while in the Add-on group, more patients discontinued due to intolerance (49.0%). Furthermore, the combined benzodiazepine dose (in diazepam equivalent values) in the Switch group decreased from 8.5 ± 5.8 mg to 3.9 ± 4.9 mg after 1 month, and 46.2% of the patients successfully withdrew from the benzodiazepines.

Another retrospective cohort study of patients with insomnia and comorbid psychiatric disorders included 90 patients who took benzodiazepines and 57 patients who took Z-drugs before the initiation of lemborexant treatment [56]. Among those, 58.9% of those who switched benzodiazepine and 63.2% of those who switched Z-drug were considered responders who showed improvements in insomnia symptoms based on the Clinical Global Impression improvement scale.

## Discussion

In this systematic review, we comprehensively extracted and reviewed clinical trials of suvorexant and lemborexant for patients with insomnia comorbid with psychiatric disorders. Most of the studies identified were cohort trials investigating patients treated with DORAs, with limited available studies of placebo-controlled RCTs.

The RCTs completed to date showed no difference in efficacy between DORAs and placebo, possibly due to the robust placebo effect and the sample size [46]. The differences from baseline in the active treatment group were similar to those observed in the phase 3 studies on primary insomnia; there is no evidence to suggest that the efficacy of DORAs for patients with comorbid psychiatric disorders is less than that for patients with primary insomnia. Seven additional RCTs are currently ongoing with suvorexant and lemborexant (Table 3) [59–65], three of which involve insomnia associated with substance use disorders (NCT05458609 [59], NCT03897062 [60], and NCT04287062 [61]). The role of orexin in the formation of dependence on substances and substance use has been suggested in nonclinical and clinical studies [66].

The results of both prospective and retrospective observational cohort studies also showed that treatment with either suvorexant or lemborexant was associated with a significantly improved sleep index in patients with insomnia who had a comorbid psychiatric disorder, without worsening the comorbid psychiatric disorders. Regarding safety, no new AEs were observed in patients with comorbid psychiatric disorders compared with traditional clinical trials, which reported similar safety findings in patients with primary insomnias.

Regarding DORA discontinuation rates, in the suvorexant cohort study (*n* = 228), the discontinuation rates were 45.0% and 24.4% in the Add-on and Switch groups, respectively [58]. Across the two groups, 36 discontinuations were due to inefficacy, and the remainder were due to AEs. In the lemborexant cohort study (*n* = 150), the discontinuation rate for patients who started lemborexant and who switched from a benzodiazepine hypnotic was 22.6% (34/150), with the most common reason for discontinuation being “Improved” (*n* = 21) [55]. One reason for the discrepancy between these two studies is the difference in the frequency of prior hypnotic medication. Another reason might be that the approved dose of suvorexant in Japan is available only at 20 mg (15 mg for elderly patients), whereas lemborexant is approved in 5-mg and 10-mg doses, meaning that the option to increase the dose of lemborexant may have influenced the improved continuation rate.

The efficacy and safety findings of the studies included in this review suggest that suvorexant or lemborexant may be recommended as an effective option for managing insomnia in those with comorbid psychiatric conditions.

### Effects of treatment of insomnia with DORAs in psychiatric disorders

That several cohort studies have demonstrated significant baseline improvements in comorbid psychiatric disorders along with improvements in insomnia in patients treated with DORAs indicates that treatment does not adversely affect the comorbid psychiatric disorder [51, 52]. Although the present study does not allow us to conclude whether DORAs ameliorate symptoms of the psychiatric disorders, administration of DORAs may benefit patients with psychiatric disorders associated with insomnia for a couple of reasons. First, DORA administration increased sleep satisfaction and reduced the burden on patients with mental illness, which may have had a spillover effect in improving mental illness severity. This is supported by two prospective cohort studies of lemborexant and suvorexant conducted with the same study design, which found that cohorts with greater changes in sleep satisfaction also experienced greater improvements in mental illness severity [50, 52]. Second, as suggested in a recent systematic review, orexin dysregulation may potentially be involved in psychiatric disorders themselves [67]. In fact, administration of orexin receptor antagonists alleviates depression, anxiety-like behaviors, and substance dependence in animal models [39, 68]. Several placebo-controlled RCTs are being conducted to examine these non-insomnia effects of DORAs [69–72].

In a phase 3 clinical trial of suvorexant, increased suicidal ideation was observed at the higher dose (30/40 mg) [42, 73]; however, suicide risk with lower doses of suvorexant is low [42, 55], no increased suicide risk has been observed with lemborexant, and the risk with both drugs is manageable by monitoring patients for signs of worsening depression or suicidal thoughts. In fact, as shown in this systematic literature review, evidence of safety in patients with depression with comorbid insomnia is accumulating. In a prospective crossover study of suvorexant, in which 15.6% (*n* = 508/3248) of the study population had depression, only one case of worsening depression was observed as an adverse effect of treatment [57]. In addition, two prospective interventional studies of suvorexant and lemborexant by Kishi et al. included 49.1% (*n* = 28) and 21.4% (*n* = 12) of patients with depression and major depression, respectively, at baseline, and did not observe any treatment-related worsening of depression [50, 52]. Therefore, DORAs appear safe for patients with insomnia as a comorbid condition of psychiatric conditions, as no deterioration of comorbid depression was observed with DORA treatment. Nonetheless, since the sample sizes in the studies evaluated were generally small, larger RCTs are needed to acquire robust results.

### Safety of DORAs in psychiatric disorders

In the suvorexant post-marketing study, the incidence of adverse drug reactions (ADRs) was higher in those with psychiatric disorders than in those without. This trend was independent of the type of comorbid psychiatric disorder [57]. Frequent ADRs included somnolence, insomnia, headache, and dizziness, which have been observed in previous clinical trials, suggesting that sleep medication-related ADRs may be more likely to occur in patients with psychiatric comorbidities. The nature of comorbid psychiatric disorders may be a contributing factor in the increasing frequency of ADRs. Furthermore, many patients were prescribed psychotropic medications, and the concomitant use of a drug that affects the nervous system may have caused nervous system ADRs. Twenty of the 144 patients with psychiatric disorders had AEs of insomnia [57], and it is possible that the drug was not sufficiently effective to treat insomnia in these patients. Therefore, careful administration and patient monitoring are needed.

Although drug treatment of insomnia should be short term, patients with comorbid psychiatric disorders often have recurrent psychiatric illnesses and treatment of insomnia can be long term. The studies reviewed here had a ≤ 6-month duration, and the safety of DORAs over a longer period of time is unknown in this population.

On the one hand, dependence and tolerance are concerns with long-term use of sleeping medications. In a prescribing analysis of BZRAs in Japan, the dose increased with the length of treatment period [73]. On the other hand, there is no clear evidence of dependence or tolerance with DORAs. In a phase 3 clinical trial in patients with insomnia, no withdrawal symptoms or rebound insomnia were observed after withdrawal of the drug after up to 12 months of medication [74, 75]. Further studies are required to evaluate long-term safety in patients with comorbid psychiatric disorders.

Of note, obstructive sleep apnea (OSA) may contribute to the development of chronic insomnia, so patients should also be assessed for the possibility of OSA. It has also been reported that the prevalence of OSA is higher in patients with psychiatric disorders [76], making this particularly important in this patient group.

### Should insomnia comorbid with psychiatric disorders be treated as a disorder independent of psychiatric symptoms?

Recently, the term “secondary insomnia” has been eliminated from the DSM-5 and other guidelines as insomnia can persist even after the primary symptoms of psychiatric disorders have been resolved. Insomnia is now regarded as a distinct disorder independent of psychiatric disorders. Based on this concept, treatment with insomnia medications should be considered for insomnia symptoms that are not sufficiently improved by psychiatric medications.

Insomnia associated with psychiatric disorders is often treated with drugs that have a strong sedative effect on the underlying disorder, which can effectively address hyperarousal and sleep disorders that commonly occur in the acute phase of a psychiatric condition [22]. However, they are not approved for insomnia and have not been rigorously studied for insomnia comorbid with psychiatric disorders. Furthermore, the use of sedative medications for psychiatric disorders presents several challenges. First, the effects of psychiatric medications on sleep can result in excessive sedation during the day, leading to reduced daytime functioning and quality of life. This is particularly true during the maintenance phase of treatment when symptoms of the primary illness have abated and the sedative effects of medication initiated during the acute phase have become more prominent. Even taking medication before bedtime may impair daytime functioning if the clinical effects persist beyond the desired duration. In addition, sedating psychiatric medications may be associated with specific adverse effects. For example, although quetiapine, clozapine, and olanzapine can influence sleep by their antihistamine effects [21], they are associated with weight gain and elevated blood lipids [77–80]. These safety issues may prevent the administration of sufficient doses of psychiatric medications to improve insomnia symptoms, further preventing the adequate management of insomnia. According to the findings of this review, suvorexant and lemborexant showed no clear adverse effects on psychiatric disorders comorbid with insomnia. In addition, DORAs are expected to have a low dependence due to their differential mechanism of action from older benzodiazepine hypnotics, making them a promising treatment option for insomnia comorbid with psychiatric disorders.

Finally, the use of DORAs in the treatment of chronic insomnia has already been discussed in other reviews [44]. Briefly, based on a network meta-analysis of phase 3 trials, the recommended strategy is to begin with 5 mg of lemborexant and increase to 10 mg if efficacy is insufficient. The network meta-analysis showed that lemborexant 5 mg is superior to suvorexant 15 or 20 mg at 1 week of treatment in subjective time to sleep onset, and 10-mg lemborexant is more effective than 5-mg lemborexant in sTST and subjective wake time after falling asleep, though 10-mg lemborexant carries an increased risk of discontinuation due to AEs and somnolence. In clinical trials, somnolence was the most common AE observed in subjects treated with suvorexant or lemborexant [41, 42, 81]. Although infrequent, nightmares, abnormal dreams, and sleep paralysis have also been reported with DORA treatment [41, 42, 78]. Because most of these events were mild-to-moderate in severity and rarely resulted in treatment discontinuation, it is advisable to inform patients in advance that these AEs may occur as a result of DORA therapy. As data emerge on DORA use in the context of psychiatric conditions, we expect to learn more about specific AE scenarios to consider when treating this patient population with this class of drugs.

### Switching from benzodiazepines to DORAs

In this review, we found a few small-scale studies confirming that suvorexant and lemborexant are efficacious and safe after switching from a BZRA or when used as an add-on therapy to a BZRA [48, 58]. For patients who have had problems with long-term BZRA use, including dependence, tolerance, cognitive impairment, and falls, switching to a DORA can be considered. The method of how to initiate DORA treatment in patients receiving BZRAs should take into account the abuse liability of the BZRA, the history and dose of the BZRA, and the user’s willingness to switch. Direct switching may be possible in patients using drugs with low abuse liability who are dissatisfied with the drug and willing to switch [82, 83]. However, for patients using a BZRA with high abuse liability, long-term users, and high-dose users, starting a DORA as an add-on while gradually tapering the previous drug may be advisable, as demonstrated in the study by Hatano et al. [58]. Because insomnia is exacerbated by anxiety, it is important to achieve sleep continuity through a combination of tapering and add-on therapy to effectively treat insomnia and increase patient confidence in their ability to sleep. In general, the available data on switching indicate that patients with insomnia and comorbid psychiatric symptoms may benefit from switching to, or adding on, a DORA if BZRA treatment does not sufficiently manage symptoms.

### Limitations

Because no large, randomized, double-blind trials were included in this systematic review, it was not possible to verify efficacy compared with placebo in terms of sleep improvement. Similarly, because of the limited number and short-term nature of studies identified, there were insufficient data available to draw conclusions on improvements in sleep for each comorbid psychiatric disorder, the impact of DORA treatment on the symptoms of the psychiatric disorders, or the long-term effects of such treatment. Moreover, results that were posted only on clinicaltrials.gov (e.g., NCT02527564 [47]) and not published in a peer-reviewed journal should be interpreted with caution. To elucidate these issues, larger-scale prospective observational studies are needed. In the treatment of insomnia comorbid with psychiatric disorders, it is also necessary to examine the compatibility of DORAs with drugs for psychiatric disorders, which will be verified through retrospective studies.

## Conclusion

In patients with psychiatric disorders, sleep deprivation is a significant burden, regardless of the type of psychiatric disorder. CBT-I is the standard treatment option and—if CBT-I cannot be used or is ineffective—a variety of drug treatment options are available. DORAs, with their unique mechanism of action, may be reasonable choices. Although there is a lack of Level 1 evidence on efficacy in patients with comorbid psychiatric disorders, evidence suggests that DORAs have a tolerable safety profile. Further studies—particularly those that are long term—are needed, and the results of the ongoing RCTs are eagerly anticipated.

## Supplementary information

Supporting information

## Data Availability

All data analyzed for this review are available in this document and supporting information.
