# Supplementary material for: Orexin receptor antagonists in the treatment of insomnia associated with psychiatric disorders: a systematic review
Source: Transl Psychiatry. 2024 Sep 14;14:374. doi: 10.1038/s41398-024-03087-4 (PMC11401906; doi:10.1038/s41398-024-03087-4)

**Supporting Information**

**Table S1. Search strategy**

**Figure S1. Identification of studies via databases**LEM, lemborexant; SUV, suvorexant

**Figure S2. Additional identification of new studies via databases**LEM, lemborexant; SUV, suvorexant

**Table S1. Search strategy**

| **Database** | **Search terms** | **Hits** |
| --- | --- | --- |
| PubMed | (lemborexant OR suvorexant) AND (Psychiatric OR "Neurodevelopmental disorder*" OR "Schizophrenia" OR ("Bipolar" OR "Bipolar disorder*") OR ("Depressive disorder*" OR depression) OR "Anxiety disorder*" OR ("Obsessive-compulsive disorder*" OR "Obsessive-compulsive related disorder*") OR ("Trauma" OR "Trauma related disorder*") OR ("Stressor related disorder*" OR "Stress disorder*" OR "Stress related disorder*") OR "Dissociative disorder*" OR "Somatic symptom*" OR ("Feeding and eating disorder*" OR "Feeding disorder*" OR "Eating disorder*") OR "Elimination disorder*" OR "Sleep-wake disorder*" OR "Sexual dysfunction*" OR "Gender dysphoria" OR "Disruptive disorder*" OR ("Impulse control" OR "Impulse control disorder*") OR "Conduct disorder*" OR ("Substance use" OR "Substance-related disorder*" OR "Use disorder*") OR "Addictive disorder*" OR "Neurocognitive disorder*" OR "Personality disorder*" OR "Paraphilic disorder*" OR "Mental disorder*") | 148  (2023/1/19) |
| Embase | (lemborexant OR suvorexant):ti,ab,kw,de AND (Psychiatric OR "Neurodevelopmental disorder*" OR "Schizophrenia" OR ("Bipolar" OR "Bipolar disorder*") OR ("Depressive disorder*" OR depression) OR "Anxiety disorder*" OR ("Obsessive-compulsive disorder*" OR "Obsessive-compulsive related disorder*") OR ("Trauma" OR "Trauma related disorder*") OR ("Stressor related disorder*" OR "Stress disorder*" OR "Stress related disorder*") OR "Dissociative disorder*" OR "Somatic symptom*" OR ("Feeding and eating disorder*" OR "Feeding disorder*" OR "Eating disorder*") OR "Elimination disorder*" OR "Sleep-wake disorder*" OR "Sexual dysfunction*" OR "Gender dysphoria" OR "Disruptive disorder*" OR ("Impulse control" OR "Impulse control disorder*") OR "Conduct disorder*" OR ("Substance use" OR "Substance-related disorder*" OR "Use disorder*") OR "Addictive disorder*" OR "Neurocognitive disorder*" OR "Personality disorder*" OR "Paraphilic disorder*" OR "Mental disorder*"):ti,ab,kw,de | 318  (2023/1/24) |
| Cochrane | (lemborexant OR suvorexant):ti,ab,kw AND (Psychiatric OR "Neurodevelopmental disorder*" OR "Schizophrenia" OR ("Bipolar" OR "Bipolar disorder*") OR ("Depressive disorder*" OR depression) OR "Anxiety disorder*" OR ("Obsessive-compulsive disorder*" OR "Obsessive-compulsive related disorder*") OR ("Trauma" OR "Trauma related disorder*") OR ("Stressor related disorder*" OR "Stress disorder*" OR "Stress related disorder*") OR "Dissociative disorder*" OR "Somatic symptom*" OR ("Feeding and eating disorder*" OR "Feeding disorder*" OR "Eating disorder*") OR "Elimination disorder*" OR "Sleep-wake disorder*" OR "Sexual dysfunction*" OR "Gender dysphoria" OR "Disruptive disorder*" OR ("Impulse control" OR "Impulse control disorder*") OR "Conduct disorder*" OR ("Substance use" OR "Substance-related disorder*" OR "Use disorder*") OR "Addictive disorder*" OR "Neurocognitive disorder*" OR "Personality disorder*" OR "Paraphilic disorder*" OR "Mental disorder*"):ti,ab,kw | 71  (2023/1/19) |
| ClinicalTrials.gov | "lemborexant” OR “suvorexant” |  |
| University Hospital Medical Information Network (UMIN) | "lemborexant” OR “suvorexant” |  |


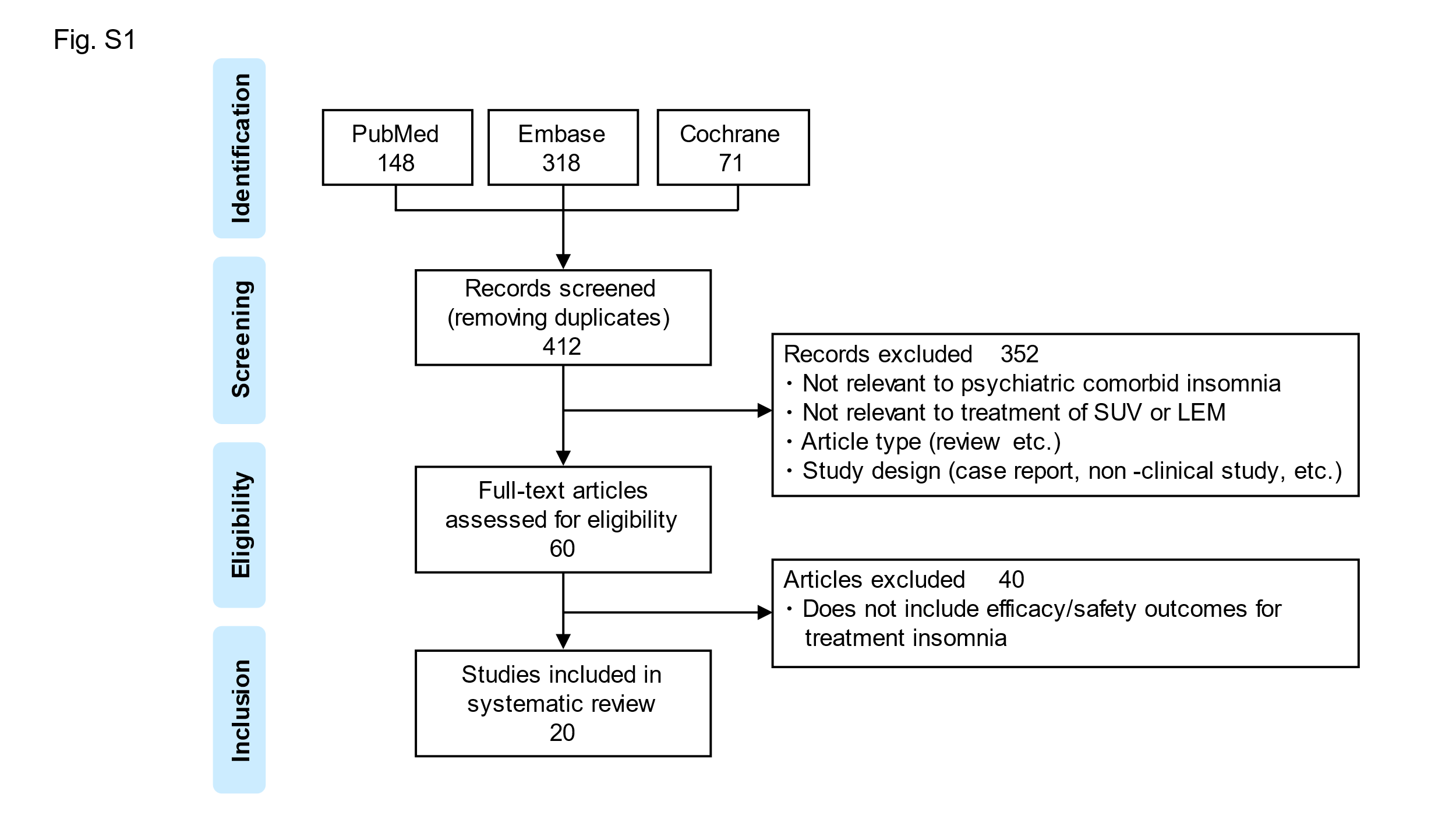


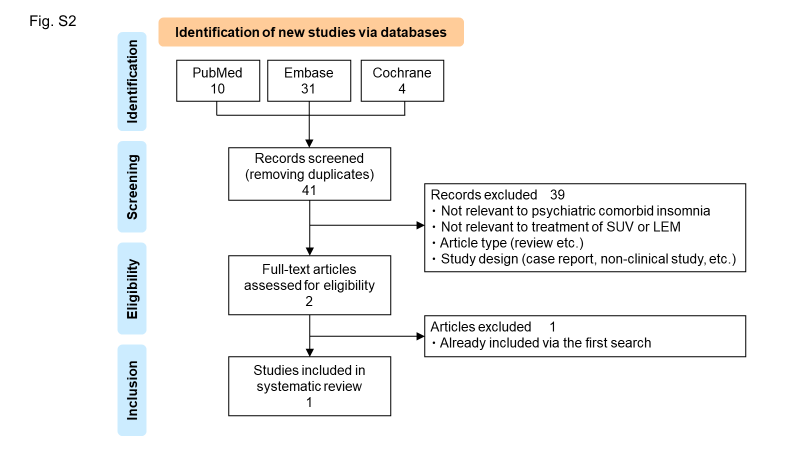

Supplement: Supplementary file 1 — Supporting information [file 41398_2024_3087_MOESM1_ESM.docx]
